# A Geospatial Semantic Enrichment and Query Service for Geotagged Photographs

**DOI:** 10.3390/s150717470

**Published:** 2015-07-20

**Authors:** Andrew Ennis, Chris Nugent, Philip Morrow, Liming Chen, George Ioannidis, Alexandru Stan, Preslav Rachev

**Affiliations:** 1 School of Computing and Mathematics, University of Ulster, Coleraine BT370QB, UK; E-Mail: cd.nugent@ulster.ac.uk; 2 School of Computing and Information Engineering, University of Ulster, Coleraine BT521SA, UK; E-Mail: pj.morrow@ulster.ac.uk; 3 School of Computer Science and Informatics, De Montfort University, Leicester LE19BH, UK; E-Mail: liming.chen@dmu.ac.uk; 4 IN2 Search Interfaces Development Ltd., 22 Forth Street, Edinburgh EH13LH, UK; E-Mails: gi@in-two.com (G.I.); as@in-two.com (A.S.); pr@in-two.com (P.R.)

**Keywords:** geospatial, Semantic, media enrichment, ontology, photograph, API

## Abstract

With the increasing abundance of technologies and smart devices, equipped with a multitude of sensors for sensing the environment around them, information creation and consumption has now become effortless. This, in particular, is the case for photographs with vast amounts being created and shared every day. For example, at the time of this writing, Instagram users upload 70 million photographs a day. Nevertheless, it still remains a challenge to discover the “right” information for the appropriate purpose. This paper describes an approach to create semantic geospatial metadata for photographs, which can facilitate photograph search and discovery. To achieve this we have developed and implemented a semantic geospatial data model by which a photograph can be enrich with geospatial metadata extracted from several geospatial data sources based on the raw low-level geo-metadata from a smartphone photograph. We present the details of our method and implementation for searching and querying the semantic geospatial metadata repository to enable a user or third party system to find the information they are looking for.

## 1. Introduction

With the increasing abundance of technologies and smart devices for creating and consuming media, such as smartphones, tablets and smart digital cameras, all containing a multitude of sensors for sensing the environment around them, it has become effortless to create vast amounts of information. In 2012, the number of smartphones globally exceeded one billion and this is expected to double by 2015 [1]. Gartner predicts that by 2015 80% of mobile handsets sold globally will be smartphones and these will outweigh PCs as the most common device to access the web [2]. The ease with which media can be captured and uploaded online results in vast amounts of information being created and stored online daily. At the time of this writing, Instagram users upload 70 million photographs a day [3]. There are also an increasing number of online information sources and tools being made publicly available, such as DBpedia [4], Flickr [5], and YouTube [6]. With this information deluge it has become increasingly time-consuming to decipher actionable information upon which informed decision-making can be based. This is particularly the case for multimedia content, such as photographs and videos where a means to better organize, categorize and make searchable the generated media is required. Users are subsequently suffering from information overload and struggling to discriminate relevant from irrelevant information. To solve this problem there is a need to have more detailed and useful metadata attached to a photograph, to facilitate and improve organization and categorization to enable relevant searches. During media capture, limited metadata is attached to the media itself, such as date/time, Global Positioning System (GPS), the camera parameters and aperture settings. Recently, with the increased use of smart digital cameras, GPS coordinates embedded in the metadata can be used for searching and categorization. In addition post-processing methods can be used by analyzing the image pixels to determine if the photograph was taken indoors or outdoors and to determine the photograph’s location based on the photometric effects of shadows cast by the sun [7]. There is, however, a specific lack of semantic geospatial information in the form of metadata. This lack of semantic geospatial metadata restricts the searching capability to the limited existing non-semantic metadata, such as the GPS coordinates with regards to geospatial metadata. The approach described in this paper, however, addresses this issue by enriching photographs with semantic geospatial metadata, which in turn allows the system to categorize and make the photographs easily searchable, based on this newly added metadata.

The remainder of this paper is organized as follows. Section 2 discusses related work; Section 3 describes the geospatial data model and the Semantic Web Rule Language (SWRL) rules used for inferring further information and geospatial relationships. It also describes the implementation of the data model; Section 4 describes the searching and querying of the semantic geospatial data repository; and Section 5 discusses the future work and conclusions.

## 2. Related Work

Geo-tagging (Geocoding) commonly refers to the process of attaching GPS coordinates to media, typically photographs. There are several ways that GPS coordinates can be attached to media. A user can manually specify the photograph location on a map, and the GPS coordinates are determined, using specialized software. More commonly the GPS coordinates are calculated automatically, by the capture device, using a GPS sensor and triangulation technologies, in the case of a smartphone or smart digital camera [8]. GPS coordinates are then stored within the photograph using the Exchangeable Image Format (EXIF) industry standard specification [8].

Reverse geocoding is the process of deriving human readable street address or place names from GPS coordinates. In order to reverse geocode media, the GPS coordinates need to be present and also a reference geospatial dataset is required. The reference geospatial dataset allows the mapping of the GPS coordinates to the address, place name or other high-level geospatial information.

The majority of related work in the media enrichment research area involves automatic image annotation based on extracted semantic features from the image pixel data.

Ballan *et al*., discuss an approach to video annotation and retrieval using ontologies and rule learning [9]. Their approach uses semantic concept classifiers and SWRL to determine what concepts are in the video and then generate annotations based on these concepts. Relevant to work in the current paper is their use of ontologies and rules to automatically determine appropriate annotations. Nevertheless, their method uses a predefined set of trained concepts to search for in a given image. This limits the annotations to only those that the system has been trained to recognize. In contrast the proposed approach in this paper to geospatial semantic annotation uses the information and concepts extracted from various publicly available datasets to then construct an ontology that enables further semantic inferences to be made based on a set of rules.

Bannour and Hudelot discuss the use of ontologies for image annotation and high-light that in order to enable complete high-level semantic annotations the use of several knowledge sources is required and inferences must be made across the knowledge sources [10].

Yi discusses the use of ontologies for the fusion of multiple geographic data sources and entity matching [11]. The work describes what information is considered relevant and useful in an ontology based on the different points of view of the different research communities. Given that this makes the process of fusing several ontologies together challenging, the paper proposes a graph model based ontology method to facilitate fusion of multiple geographic ontologies. The approach matches entities, however it lacks the ability to determine geospatial relationships between the entities, which is a particular media requirement for our geospatial model.

Lacerda *et al*., proposed an approach to improve the quality of geotags by propagating the geotags across similar photographs [12]. Their research was primarily focused on personal photograph collections, where some of the photographs were tagged and others were missing tags but were taken in the same region. To tag the non-tagged photographs, their system searches for tagged photographs that are temporally close within the cluster, based on features extracted from the photograph pixel data. Their system then propagates the relevant tags to the untagged photograph. One of the issues they discovered is that when a photograph is taken from a vehicle moving at speed, e.g., airplane or high speed train, then the photographic process will have occurred over a large spatial distance from the point that the capture process started. However if the photograph is being clustered by GPS, it could actually be located a large distance from where the GPS coordinates were captured [12]. To overcome this, Lacerda *et al*. proposed to also capture the speed information from the GPS sensor. The speed information is used to determine if there is a geo-reference inconsistency between two photographs by calculating if the user reaches the location of the second photograph in the time it takes to capture the photograph [12]. Lacerda *et al*. validated their results by comparing the geotags that have been propagated with the real location of the non-georeferenced photographs. Their results indicated that their system achieved good results with a precision of 97.08% and a recall of 73.97% [12].

FollowThePlace [13], a platform developed by IN2 Search Interfaces Development Ltd., allows a user to upload photographs of places and add annotations that facilitate searching and browsing the photographs taken by users of places. FollowThePlace currently reverse geocodes the geo-tagged photographs, however, they only go to the granularity level of the city level and not the precise location. This makes it difficult to create string relationships between the media and allow reasonable inferences to be made.

Taking into consideration the previous research carried out in the area of geospatial semantic enrichment of photographs, it is clear there are a number of research gaps. The research in this paper focuses on the modeling of high-level geospatial information that has been extracted from several geospatial data sources, based on the GPS coordinates from the photograph that is being enriched. The research in this paper also looks at an approach to how the generated geospatial data model can be queried and searched, therefore enabling better searching and organization of photographs.

## 3. Geospatial Data Modeling

In our previous work, we have developed a system, known as MediaPlace, to extract geospatial information from multiple geospatial data sources, including Geonames, DBpedia, Google Places, and OpenStreetMaps [14,15]. The types of information extracted from these datasets are latitude, longitude, place name, place feature type and feature description, city, country, and elevation. The system, developed in our previous work uses the GPS coordinates in a photograph to query the datasets and extract the different items of information and fuse the result sets together. This fusion process combines duplicates that may appear in the multiple datasets or combinations of two places that are similar enough to be assumed the same place, based on given criteria [14,15]. The criterion of the fusion process involves initially checking if any of the extracted Point Of Interests (POI) have a reference to each other, such as a “sameAs” attribute. This is in the form of a “sameAs” attribute with a URI to the other POI. If it does have an associated “sameAs” attribute that links to the second POI, the system trusts this and classifies them as the same. If no “sameAs” attribute is found, then the system checks to see how far apart the two POIs are from each other. The distance threshold will vary depending on the feature type that the POIs have. If a POI has a feature type of “statue” then the spatial footprint will be relatively small. Whereas a POI with a feature type of “building” will have a relatively large spatial footprint. This also means that the POIs have to have the same feature type if they are to be classified as being the same.

In the following Sections we discuss an extension to our work. This involves modeling the data extracted from several geospatial data sources and automatically inferring the relationships between the data and the media.

### 3.1. Data Model

Once the geospatial information has been extracted and fused, it then needs to be analyzed and interpreted so that an understanding of how the data relates to the media can be established in an automatic way. To do this, the data needs to be modeled such that inferences can be made across the data, thus enabling relationships between the extracted data and the media to be determined, for example, storing the data and relationships in a semantic description format, such as Resource Description Framework (RDF). The model needs to be able to store links back to the original data source in order to enable verification and provenance of the data. Given that some of the data sources are part of the linked data cloud, this link to the original data source can be used by applications as an entry point to the linked cloud. Applications can then further follow the linked cloud links and gather more information beyond the scope of the extracted geospatial data.

**Figure 1:**
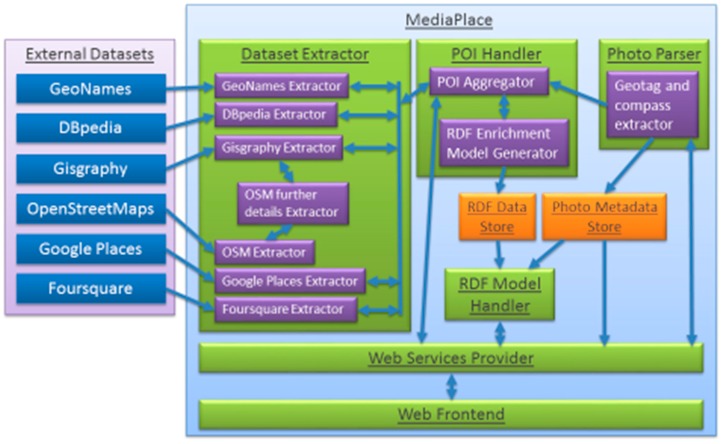
Diagram showing MediaPlace architecture with the external datasets on the left and our photograph enrichment system on the right. It shows each component of the system and how each component interconnects.

For the data model to handle all the criteria described above we choose to model the data in an ontology. This has the added benefit of being able to infer further relationships and also to reason over the data when executing a query. To handle the data model, the MediaPlace architecture was modified, as shown in Figure 1, so that in the POI Handler layer contains the RDF Enrichment Model Generator component, which creates the sets of triples representing the extracted objects and their attributes, such as latitude, longitude, place name, and place feature (building, statue, *etc*.) [14,15]. A POI is a significant location that may be of interest and, in relation to MediaPlace, is usually a building, statue, or business location. These triples represent subject-predicate-object. For example *POIA hasName Belfast City Hall* and *Photograph isLookingAt POIA*. During the semantic metadata repository population phase, predefined attributes are calculated, such as the distance to each POI from the photograph. This pre-calculation enables faster processing in our semantic geospatial metadata repository later on, particularly when searching. SWRL rules are also then run against the data stored in the semantic metadata repository to determine further information through inferences for example what POIs the photograph is looking at or the direction relationship each POI has to the photograph. This then enables searching, such as show all POIs to the south of a given photograph, or show all photographs to the east of a given POI.

### 3.2. Implementation of Data Model

**Figure 2:**
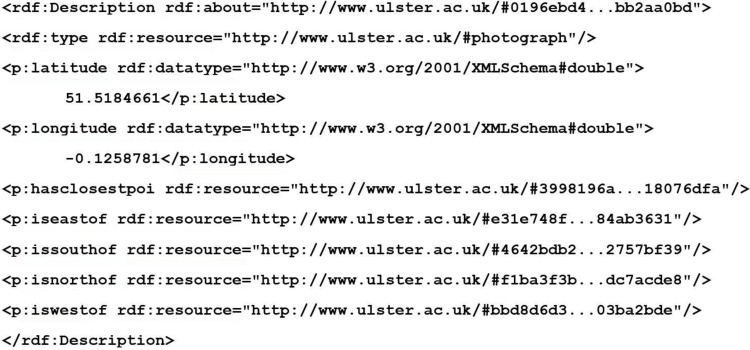
Snippet of RDF XML created for a photograph by MediaPlace. The 256 bit SHA hash has been abbreviated for improved presentation clarity.

The semantic geospatial metadata of each photograph is generated by instantiating the ontological geospatial data model, which is assigned a unique id so that it can be identified. The id is a 256 bit Secure Hash Algorithm (SHA) hash generated from the latitude, longitude, and compass of the photograph. This ensures that every photograph will have its own unique id, however, it also ensures that the same photograph is not processed twice. In other words, the same place and direction, as the geospatial model for that location has already been computed. Figure 2 shows an example of the semantic geospatial metadata in RDF XML format created for a photograph.

**Figure 3:**
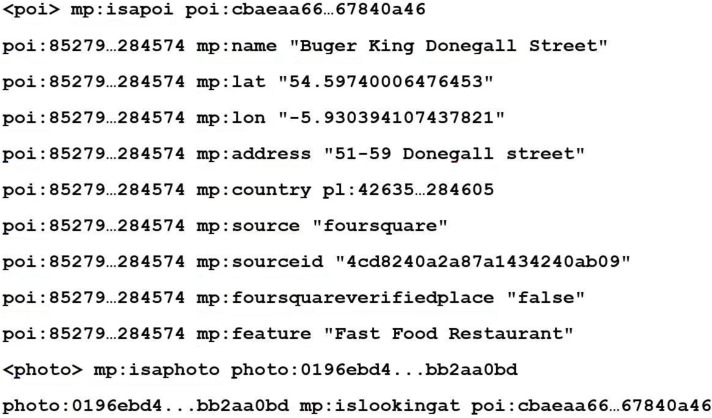
Snippet of RDF, for a POI, created by MediaPlace. The 256 bit SHA hash has been abbreviated for improved presentation clarity.

For each POI extracted from the datasets a “poi” node is created which is identified with a 256 bit SHA hash. This SHA hash is created from the place name, latitude and longitude. This ensures that each POI node has a unique identifier that each attribute can be linked to. It also enables checking if identification of the POI has already been added to the semantic geospatial metadata repository, therefore stopping it from being added twice. Figure 3 shows a short example of some of the triples that are created for a given POI.

The administration hierarchy of each of the POIs is also modeled, such as the city, country and continent that the POI is located within. This is done by using the associated city or country information extracted from the POIs. MediaPlace then searches against the Geonames database to obtain the remaining hierarchical information. For example: if the city name exists, the country that this city is located in can be queried from the Geonames database. The continent that contains the country can then in turn be queried. MediaPlace cannot, however, solely rely on Geonames to provide an accurate country or city name because its data is collected from a variety of sources and errors can appear. To address this, a cross-reference with the other datasets is done. For example if results are obtained from Google Places for the photograph, then country and/or city information is obtained and checked if there is a match with the other country information from the other POIs. If a new country is introduced into the semantic geospatial metadata repository that currently does not exist, then there is a conflict and it needs to be resolved. This is done by looking, not only at the frequency counts of the country names, but also by looking at higher or lower levels of the administration hierarchy. To determine the most common country from the POIs a simple frequency-voting algorithm on the country name is used, were the country with the most votes from the POIs, is the most likely country name for the given location.

### 3.3. SWRL Rules

SWRL is a language used to construct inference rules, which are run against triples to try and infer more semantic relationships. This is particularly useful for MediaPlace given that it enables the system to infer relationships between a given photograph and many POIs that are not explicitly there in the extracted information. Nevertheless, they can be inferred using the rules and the extracted data.

**Figure 4:**
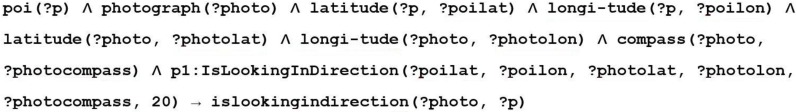
Example SWRL rule used by MediaPlace to determine what POIs the photograph is looking in the direction of.

SWRL rules are applied to the semantic geospatial data repository for collected photographs to infer further information and relationships, in particular that of the POIs of the photograph. Due to the complexity of geospatial calculations several custom functions were developed, called built-ins, for example to calculate compass bearing between two GPS points, distance between two GPS coordinates and POI name similarity. These built-ins then supply values back to the rule, which can be used for comparisons or in the result of the rule. An example is shown in Figure 4, where one of the rules is to calculate if the POI is in the direction the photograph was taken. This coupled with the distance from the photograph, means that when the system queries the semantic geospatial metadata repository to determine what the photograph is looking at, these values are already calculated and so the query is much more simplified and also computationally simpler. The result from the rules gets added back into the semantic geospatial metadata repository and so is adding additional semantic context to the photograph, such as how far away the POIs are and in what direction, *etc.*

## 4. Querying and Searching the Semantic Geospatial Metadata Repository

To make use of our geospatial data model we have implemented a web service that enables third party systems or a user to query and search the semantic geospatial metadata repository for the pieces of information that are of most interest to them. To achieve this searching capability, the geospatial annotations for photographs are stored in a semantic repository. Virtuoso was chosen for the semantic repository because it is a popular semantic repository. Our reasoning for using a semantic repository was because our model is based on triples, which describe a semantic meaning of the data. Therefore, Virtuoso enables us to retain this model and combine the models from many photographs together, however, importantly a web service can be developed on top to abstract away the underlying structure and provide a simple Application Programming Interface (API) for querying the semantic geospatial metadata.

The web service provides two main services. The first enables a photograph to be enriched and the enrichment metadata to be stored for later querying. This is achieved by supplying the GPS coordinates of the photograph and an optional compass heading. This is enough for MediaPlace to query the external datasets and create the enrichment semantic geospatial metadata. For example, if a user wishes to enrich a photograph at the coordinates 54.597N, −5.930E and a compass of 174° and they want the semantic geospatial metadata to be returned, the query for this service would be as follows:

http://127.0.0.1/api/enrich/?location=54.5970788,-5.9301246&compass=174&returnmodel=true

In the above *location* is the GPS coordinates of the photograph. *Compass*, is optional, being the heading the photograph was taken at, relative to true north. *Returnmodel*, is optional, being a Boolean flag indicating if the model should be returned to the calling application.

The second of the services provided enables querying of the semantic geospatial enrichment repository. This enables a user to specify a location of interest, an optional compass heading relative to true north, optional distance in meters, optional features, and an optional free text string.

To facilitate a user in knowing what features are available a service is provided that returns all the features that are currently stored in the repository. This forces a user to search on only the types of data that are actually in the repository, therefore helping to direct them to relevant information. For example if a user wishes to search for a hotel with a free text term inn, and within 500 m of a photograph at the GPS point 54.597N, –5.930E and a compass heading of 174°, the query for this service would be as follows:

http://127.0.0.1/api/search/?location=54.5970788,-5.9301246&compass=174&distance=500&features=hotel&searchtext=inn

The above API call could be the scenario where a user is using a photograph to search for a hotel. The photograph could be of a famous monument and they are looking for a hotel within walking distance of the monument. Our search algorithm can also be used in reverse, in other words to find photographs in a particular location, with particular features.

A result set is returned from the search API call containing the details of the POIs that match the supplied criteria. This information can then be displayed to the user, in a user friendly and readable way, to provide them a greater understanding about the photograph and where it was taken. An example result would be as follows:

**Figure 5:**
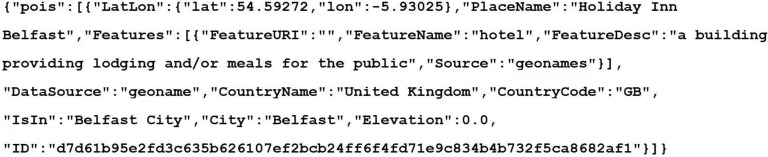
JavaScript Object Notation (JSON) object returned from API search query.

Figure 5 shows the JSON object returned from the API query. It contains one POI and its attributes, such as place name, its features, and feature description. This can then be used by a smartphone app, for example, to display the relevant information back to the user.

Information about related streets and open areas or parks can also be returned if the search criteria have selected this type of information to be returned.

### Integration

**Figure 6:**
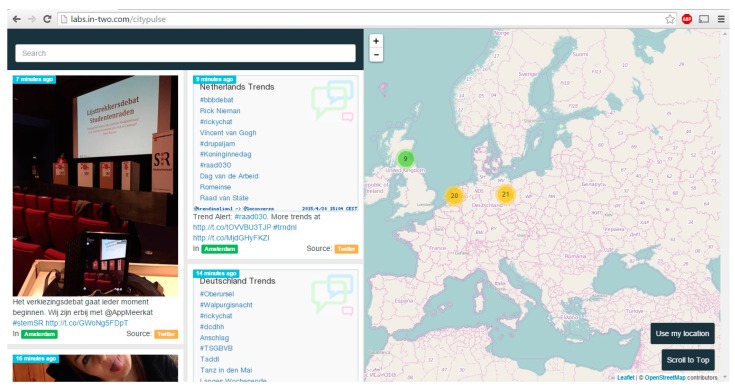
Screenshot of CityPulse app showing the user interface [16].

**Figure 7:**
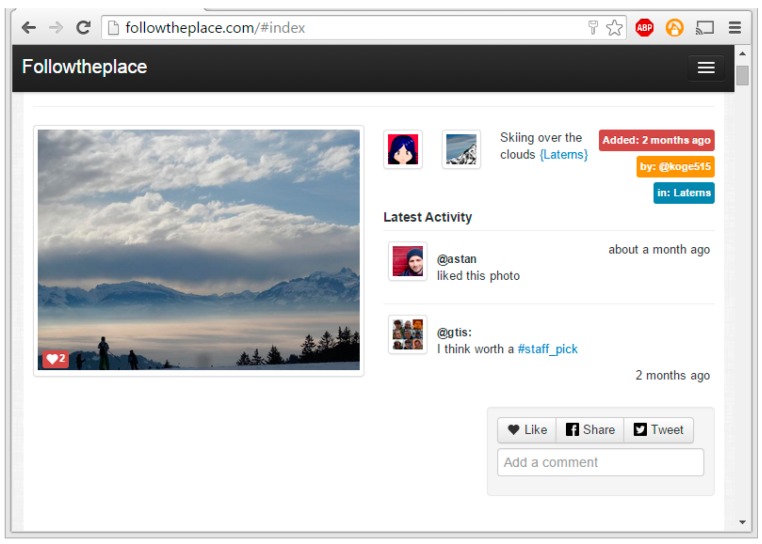
Screenshot of FollowThePlace app showing the user interface [13].

IN2 Search Interfaces Development Ltd. provides a number of services that enable organizing, searching and displaying multimedia. Two of their services, FollowThePlace and CityPulse, focus on photographs and places. Screenshots of these services are shown in Figure 6 and Figure 7.

IN2 are using the API developed in the current work by integrating it with CityPulse and FollowThePlace services to enhance the searching and categorization of digital media that these two services provide. Our API is being integrated into the backend system of CityPulse to enrich the search indexing and the categorization of the photographs with our semantic geospatial metadata. This integration will therefore allow a user to search on the semantic geospatial enrichments to better find photographs and information that is of interest to them. The semantic geospatial metadata will also be used for suggesting similar photographs to the user, by matching photographs on geospatial features, such as both are looking at the same statue.

## 5. Future Work and Conclusions

To verify that MediaPlace is producing correct and relevant semantic geospatial annotations, we propose to run a subset of geotagged photographs from the Yahoo Flickr Creative Commons 100 M dataset [17]. As per the Yahoo Data Sharing Agreement, this dataset will solely be used for the academic research purposes of producing publishable results to validate our research.

This dataset has been compiled by Yahoo from Flickr’s collection of photographs and videos. The dataset does not contain the original photograph pixel data, however, is a comma-separated file containing the metadata of the photograph, such as the geotag coordinates and user created tags. One of the metadata attributes is a URL to the original photograph in Flickr, which can be downloaded if necessary, however we will not be downloading the original image, as it is not required by MediaPlace. Since we are only interested in geotagged photographs, a subset of photographs is taken that meet the following criteria: must be geotagged, must have user tags, must have a description and optionally have machine tags.

Our justification for this selection criteria is as follows:

• Geotagged: MediaPlace requires GPS coordinates. Although this dataset does not contain the compass heading, MediaPlace will still work without it. We just will not be able to determine the *islookingat* attributes.
• User tags: these are what will be compared to the MediaPlace tags. A string similarity will be used as the metric.
• Description: a comparison of the words in the description will also be carried out.
• Machine tags: if these are available they will be compared to the MediaPlace tags. A string similarity will be used as the metric.

Since MediaPlace uses online APIs, there are query restrictions imposed by the providers of the APIs, so we initially intend to further reduce the photograph set to 2500 photographs for the first run.

The statistics that will be gathered are what percentage of photographs MediaPlace correctly determines geospatial annotations for and the accuracy of the geospatial annotations. This will be achieved by performing a string comparison between the geospatial annotations created by MediaPlace and the user tags, machine tags, and description from the Flickr dataset.

The precision of MediaPlace at determining the main POI the photograph is looking and in addition the precision of the features MediaPlace assigns to the photograph will be determined. The precision of the place names assigned by MediaPlace will also be compared with the user tags, machine tags and description from the dataset to ascertain the accuracy of MediaPlace in determining where the photograph has been taken. This will include the accuracy of the hierarchy of places, for the photographs where the tags have this data. For example: Donegall Square is in Belfast City, Belfast City is in Northern Ireland, Northern Ireland is in the UK, the UK is in Europe.

In the future the dataset will be increased from 2500 to 5000 photographs in a second run, to see if similar statistics are gathered. 5000 photographs will produce a sufficient number of results to generate statistics, which will show if MediaPlace is accurate at determining sensible geospatial annotations for a geotagged photograph.

In conclusion this paper presents the details of a semantic geospatial data model, which provides a way to enrich photographs with rich semantic metadata extracted from several datasets. In addition, the development of SWRL rules to infer relationships between the extracted information and a photograph have been presented. The development of an API to enable querying and searching of the semantic geospatial enrichment metadata associated to a photograph has also been presented. The API demonstrates that the semantic geospatial enrichment metadata can be used to find and discover geospatial information about a photograph. The API also demonstrates the ability to search and discover photographs based on geospatial features.

This paper also presents a discussion of how we propose to use the Yahoo Flickr Creative Commons 100 M dataset to gather statistical results that will validate if MediaPlace is producing reasonably accurate and precise geospatial enrichment results.
